# High fat diet impairs the function of glucagon-like peptide-1 producing L-cells

**DOI:** 10.1016/j.peptides.2015.06.006

**Published:** 2016-03

**Authors:** Paul Richards, Ramona Pais, Abdella M. Habib, Cheryl A. Brighton, Giles S.H. Yeo, Frank Reimann, Fiona M. Gribble

**Affiliations:** Metabolic Research Laboratories and MRC Metabolic Diseases Unit, WT-MRC Institute of Metabolic Science, Addenbrooke's Hospital, Cambridge CB2 0QQ, UK

**Keywords:** GLP-1, Enteroendocrine, L-cell, High fat diet

## Abstract

Glucagon-like peptide-1 (GLP-1) acts as a satiety signal and enhances insulin release. This study examined how GLP-1 production from intestinal L-cells is modified by dietary changes.

**Methods:**

Transgenic mouse models were utilized in which L-cells could be purified by cell specific expression of a yellow fluorescent protein, Venus. Mice were fed on chow or 60% high fat diet (HFD) for 2 or 16 weeks. L-cells were purified by flow cytometry and analysed by microarray and quantitative RT-PCR. Enteroendocrine cell populations were examined by FACS analysis, and GLP-1 secretion was assessed in primary intestinal cultures.

**Results:**

Two weeks HFD reduced the numbers of GLP-1 positive cells in the colon, and of GIP positive cells in the small intestine. Purified small intestinal L-cells showed major shifts in their gene expression profiles. In mice on HFD for 16 weeks, significant reductions were observed in the expression of L-cell specific genes, including those encoding gut hormones (*Gip*, *Cck*, *Sct*, *Nts*), prohormone processing enzymes (*Pcsk1*, *Cpe*), granins (*Chgb*, *Scg2*), nutrient sensing machinery (*Slc5a1*, *Slc15a1*, *Abcc8*, *Gpr120*) and enteroendocrine-specific transcription factors (*Etv1*, *Isl1*, *Mlxipl*, *Nkx2.2* and *Rfx6*). A corresponding reduction in the GLP-1 secretory responsiveness to nutrient stimuli was observed in primary small intestinal cultures.

**Conclusion:**

Mice fed on HFD exhibited reduced expression in L-cells of many L-cell specific genes, suggesting an impairment of enteroendocrine cell function. Our results suggest that a western style diet may detrimentally affect the secretion of gut hormones and normal post-prandial signaling, which could impact on insulin secretion and satiety.

## Highlights

• Long term dietary changes impair function of the gut endocrine system.
• High fat diet impairs nutrient-triggered GLP-1 release from murine small intestine.
• L-cells from HFD-fed mice have reduced expression of many L-cell-specific genes.

## 1 Introduction

Hormones from the gut control food intake and insulin release as well as intestinal motility and secretion [1]. The post-prandial rise in the plasma concentration of GLP-1 signals to the brain that food has arrived in the gastrointestinal tract and to the pancreas that glucose is being absorbed. GLP-1 brings about the sensation of satiety together with the enhancement of insulin release [2]. As dietary habits have changed and people now consume more fat and sugar, this raises concerns about whether dietary intake affects the release of gut hormones, and whether this in turn might contribute to rising levels of obesity and diabetes.

GLP-1, like other gut hormones, is released from enteroendocrine cells (EECs) located in the intestinal epithelium. EECs have a life span of only about 5 days, and are constantly replenished from stem cells in the intestinal crypts. Depending on their position along the gastrointestinal tract, EECs exhibit characteristic hormonal signatures. In the duodenum, for example, there is a high number of K-cells producing GIP, whereas in the distal ileum and colon there are more L-cells expressing GLP-1 and PYY [3]. A few published studies suggest that the number of EECs can be altered by environmental stimuli. In intestinal organoid cultures in vitro, for example, the number of L-cells is increased by exposure to short chain fatty acids [4], and in vivo, elevated L-cell numbers have been observed in germ-free mice [5], and in rodents on a high-fiber diet [6]. How EECs are affected by dietary exposure is an area of great interest. If it were possible to increase L-cell number, this could lead to increased GLP-1 release and improved glucose tolerance and satiety.

Surgical models have been used to investigate how EEC numbers are affected by exposure of different gut segments to luminal nutrients. In rats, gastric bypass surgery has been reported to result in an increase in L-cell numbers in the roux (alimentary) and common limbs [7], [8]. In both cases, it was found that the increase in L-cell numbers was attributable to mucosal hypertrophy, but that there was no change in L-cell density. EECs also developed normally in the biliopancreatic limb. These studies suggest that increased ileal nutrient exposure in the context of a chow diet results in mucosal growth and a corresponding increase in L-cell number, but that the frequency of L-cells is not altered.

It is less clear how the intestine responds to diets containing a high fat content. In high-fat diet (HFD) fed rodents, it was reported that there is a reduction in the density of cells staining for chromogranin A and GLP-1, and correspondingly reduced expression of *Gcg* and *Pyy* by PCR [9], [10]. In this study we have examined the transcriptomic and secretory properties of L-cells from mice fed on a high fat (60%), high sugar diet.

## 2 Materials and methods

### 2.1 Animals

All procedures were approved by the UK Home Office and local ethical committee of the University of Cambridge. Male GLU-Venus mice [11] on a C57Bl6 background were weighed at age 8 weeks and divided into 2 groups (*n* = 4–6 per group), each with a balanced range of body weights. For the 16-week study, they were then single housed and placed on either a standard control chow (Special Diets Services, Rat and mouse breeder and grower diet) or high fat diet (Research Diets, #D12331, containing 60% energy from fat). Mice were weighed weekly for 16 weeks. Mice on the 2-week study were treated similarly, but were not single housed. Mice were killed by cervical dislocation 2–4 h after lights on. Intestinal tissues were harvested and treated as below for flow cytometry (FACS), mRNA extraction or secretion experiments.

### 2.2 Small intestine for FACS sorting

For purification of cell populations by FACS, intestinal pieces were stripped of the outer muscle layers. Tissue was chopped into 1–2 mm pieces and digested to single cells with 1 mg/ml collagenase in calcium-free Hanks Buffered Salt Solution (HBSS). Single cell suspensions were separated by flow cytometry using a MoFlo Beckman Coulter Cytomation sorter (FL, USA). Side scatter, forward scatter and pulse width gates were used to exclude debris and aggregates, and Venus positive cells collected at ∼95% purity, alongside negative (control) cells that comprised a mixed population dominated by other epithelial cell types.

### 2.3 FACS analysis

Single cell suspensions were prepared for FACS analysis, as described previously (9). Briefly, cells were fixed with 4% paraformaldehyde (PFA) in phosphate buffered saline (PBS), permeabilised with 0.1% (v/v) Triton X-100, blocked with 10% goat serum and then incubated with primary antibody in PBS-10% goat serum at 4 °C overnight for an hour at RT. The primary antibodies used were: anti-GIP (gifted by Prof. J.J. Holst; 1:1000), anti-CCK (gifted by Prof. G.J. Dockray; 1:500) and anti-PYY (Progen, London, UK; 16066; 1:100). Cells were rinsed 3 times and then incubated with secondary antibody (Alexa-Fluor 555, 633 or 647 Invitrogen, USA; A-21435, A-21105, A-21428 or A-21070, all at 1:800). Cells were analysed using a LSRCyAn advance digital processing Flow Cytometer (Dako Cytomation, CA, USA; Fortessa (BD Biosciences)). Events with very low side and forward scatter were excluded as these are likely to represent debris, and events with a high pulse width were excluded to eliminate cell aggregates.

### 2.4 RNA extraction and qRT-PCR

Tissues used for RNA and protein extraction were washed in PBS and placed in RNAlater (Ambion, Life Technologies, Paisley, UK) and frozen until processed. Samples were homogenized in Tri-reagent (Sigma), and then treated as below. Total RNA from FACS-sorted cells prepared from GLU-Venus transgenic mice was isolated using a micro scale RNA isolation kit (Ambion). All samples were reverse transcribed according to standard protocols. Quantitative RT-PCR was performed with 7900 HT Fast Real-Time PCR system (Applied Biosystems, Life Technologies), using verified Taqman primer/probe sets supplied by Applied Biosystems. In all cases expression was compared with that of β-actin measured on the same sample in parallel on the same plate, giving a CT difference (ΔCT) for β-actin minus the test gene. Mean, standard error, and statistics were performed on the ΔCT data and only converted to relative expression levels (2^ΔCT^) for presentation in the figures.

### 2.5 Microarray

The quality of RNA samples was determined by Total RNA Pico Chip (Agilent Technologies, Stockport, UK), and only those with RIN values >7.0 selected for microarray analysis. Total RNA from the samples were amplified using the NuGEN Ovation Pico WTA system (NuGEN Technologies, Leek, Netherlands) according to the manufacturer's protocol, labeled with the NuGEN Exon and Encore™ Biotin Modules, and hybridized onto Affymetrix Mouse Gene ST 1.0 arrays. Raw microarray image data were converted to CEL files using Affymetrix GeneChip Operating Software. All the downstream analysis of the microarray data was performed using GeneSpring GX 12.1 (Agilent). The CEL files were used for both the robust multi-array average (RMA) and Probe Logarithmic Intensity Error (PLIER) analyses. After importing the data, each chip was normalized to the 50th centile of the measurements taken from that chip.

To assess the purity of L-cell sorts, each probe on the microarray data was assigned a value indicating its relative expression in L-cells vs non-L-cells (combining all microarrays from HFD and chow-fed mice), and the data were ordered to indicate the top 50 probes most enriched in the non-L-cell population. For each L-cell microarray we then calculated the geometric mean of these 50 non-L-cell probes as a measure of contamination by non-L-cells. One out of the 4 microarrays from HFD L-cells was excluded from further analysis because its mean expression of non-L-cell probes was >2 SD above the mean of all L-cell microarrays. In further analyses, the number of individual microarrays used was: L-cells on chow (3), L-cells on HFD (3), non-L-cells on chow (3) and non-L-cells on HFD (4).

### 2.6 Primary intestinal culture

Small intestinal and colonic primary cultures were prepared as previously described [11]. Briefly, mice 3–6 months old were sacrificed by cervical dislocation and the small intestine or colon was excised. Luminal contents were flushed thoroughly with PBS and the outer muscle layer removed. Tissue was minced and digested with Collagenase Type XI and the cell suspension plated onto 24-well plates pre-coated with Matrigel (BD Bioscience, Oxford, UK).

### 2.7 GLP-1 secretion assay

18–24 h after plating, cells were washed and incubated with test agents made up in 0.25 ml saline buffer containing: (in mM: 138 NaCl, 4.5 KCl, 4.2 NaHCO_3_, 1.2 NaH_2_PO_4_, 2.6 CaCl_2_, 1.2 MgCl_2_, and 10 HEPES, pH 7.4 with NaOH) supplemented with 0.1% BSA for 2 h at 37 °C. At the end of the 2-h incubation, supernatants were collected and centrifuged at 2000 rcf for 5 min and snap frozen on dry ice. Cells were mechanically disrupted in 0.5 ml lysis buffer containing (mM): 50 Tris–HCl, 150 NaCl, 1% IGEPAL-CA 630, 0.5% deoxycholic acid (DCA) and complete EDTA-free protease inhibitor cocktail (Roche, Burgess Hill, UK) to extract intracellular peptides, centrifuged at 10,000 rcf for 10 min and snap frozen. GLP-1 was measured using an electrochemiluminescence total GLP-1 assay (MesoScale Discovery, Gaithersburg, MD, USA) and results expressed as a percent of total (secreted + lysate) GLP-1 and normalized to basal secretion in response to saline measured in parallel on the same day. Chemicals were purchased from Sigma (Poole, UK) unless otherwise indicated.

### 2.8 Data analysis

Results are expressed as mean ± SEM. Statistical analysis was performed using GraphPad Prism 5.01 (San Diego, CA, USA). For GLP-1 secretion data, one-way ANOVA with post hoc Dunnett's or Bonferroni's tests were performed on log-transformed secretion data, as these data were heteroscedastic. Values were regarded as significant when *p* < 0.05.

## 3 Results

### 3.1 HFD affects the expression of gut hormone mRNAs in tissue homogenates

**Fig. 1:**
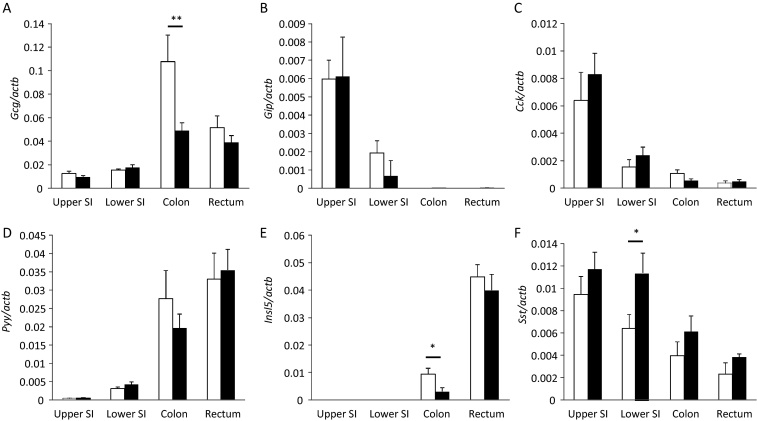
Effect of 2 weeks HFD on hormone gene expression in the gastrointestinal tract. Expression of gut hormone mRNAs was quantified by qRT-PCR in tissue homogenates from the small intestine (SI), colon and rectum of mice fed on chow (white bars) or HFD (black bars) for 2 weeks. Expression of *Gcg* (A), *Gip* (B), *Cck* (C), *Pyy* (D), *Insl5* (E) and *Sst* (F) is shown relative to that of *Actb* measured in parallel on the same samples. Columns represent the mean of *n* = 5 (chow) and *n* = 7 (HFD) samples, and the error bars represent 1 SEM. Statistics were performed on the ΔCT data (actb-gene of interest) and the data transformed by 2^ΔCT^ for illustration in the figures. *p < 0.05, **p < 0.01 by Student's *t*-test.

Two weeks on HFD resulted in reduced expression of *Gcg* and *Insl5* in colonic tissue, and a tendency toward reduced expression of *Cck* and *Pyy*, suggesting that HFD causes either a reduction in colonic L-cell density or impaired production of hormones by individual enteroendocrine cells (Fig. 1). No significant changes were observed in the expression of *Gcg*, *Cck*, *Gip* or *Pyy* in small intestinal tissue homogenates, although we did detect an increase in somatostatin (*Sst*) mRNA in the ileum of HFD-fed mice. Hormone expression was unaffected in the rectum.

### 3.2 HFD alters gut peptide production by individual L-cells

**Fig. 2:**
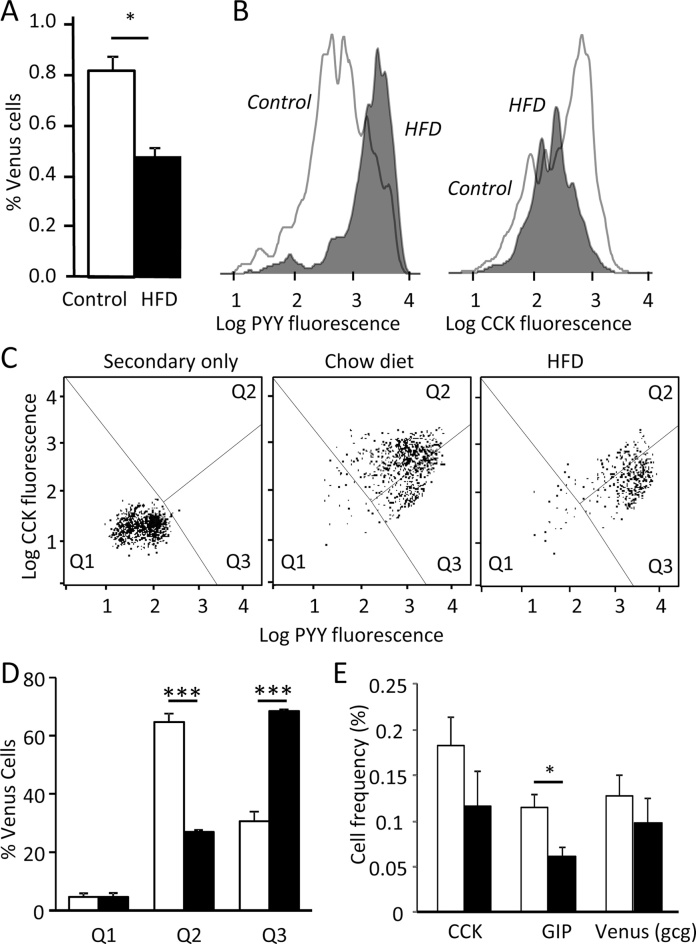
Analysis of enteroendocrine cell numbers by FACS. (A) The frequency of L-cells was reduced in the colons of mice fed on HFD for 2 weeks (black bars) compared with chow-fed controls (white bars), as assessed by counting Venus-positive cells by FACS in tissues from GLU-Venus mice. (B–D) Colonic cell suspensions, as in (A), were fixed and stained for PYY and CCK, using red and far red secondary antibodies. (B) The red/far red fluorescence intensity histogram (indicating the intensity of staining for PYY and CCK) of the Venus positive L-cell population is shown for one representative each of a chow-fed (control) and HFD-fed mouse. Increased intensity of PYY staining, and reduced intensity of CCK staining is apparent in the HFD-fed mouse. (C) CCK and PYY staining obtained as in (B) are depicted for a representative chow fed mouse without/with primary antibodies (left and center, respectively) and a HFD-fed mouse. Each dot represents an individual Venus-positive cell. (D) Mean percentages of Venus-positive cells in quadrants 1–3 (Q1, Q2, Q3) for cell suspensions analysed as in (C). (E) The small intestines of chow-fed and HFD-fed mice were analysed by FACS, as in (A, B), and the frequencies of cells positive for Venus, CCK and GIP were quantified as a percentage of the total cell number. Columns represent the mean, and error bars represent 1 SEM of *n* = 3 (colon) and *n* = 5 (small intestine). *p < 0.05, ***p < 0.001, by Student's *t*-test.

To evaluate the frequency of L-cells and their individual production of peptide hormones, we performed FACS analysis of cell suspensions from transgenic mice expressing a yellow fluorescent protein Venus driven by the *gcg* promoter, which were immunostained for different gut hormones. Quantification of Venus-labeled L-cells in different regions of the GI tract revealed that the large intestine (colon + rectum) of HFD-fed mice contained a significantly lower percentage of Venus positive L-cells than their chow-fed littermates (Fig. 2A). In large intestinal cell suspensions co-stained with antibodies against CCK and PYY, we observed a particular reduction in the number of L-cells staining strongly for CCK, and a corresponding increase in the intensity of L-cell PYY staining in mice fed on HFD for 2 weeks (Fig. 2B–D).

In the upper small intestine, the frequency of Venus-labeled L-cells was not markedly affected by the HFD (Fig. 2E). Both the frequency of GIP-stained cells (Fig. 2E), however, and the proportion of L-cells that were immuno-positive for GIP (data not shown) were reduced in small intestinal cell suspensions from HFD-fed mice. Staining for PYY and CCK were not noticeably different between the chow and HFD groups in the small intestine (Fig. 2E and data not shown).

### 3.3 GLP-1 release is altered in intestinal cultures from HFD-fed mice

**Fig. 3:**
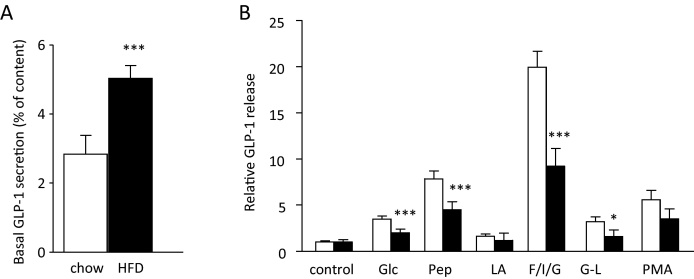
Effect of HFD on small intestinal GLP-1 release. Small intestinal cultures from mice fed for 2 weeks on chow (open bars) or HFD (black bars) were incubated for 2 h in control buffer (A), or in buffer containing 10 mM glucose (Glc), 0.5% peptone (Pep), 100 μM linoleic acid (LA), 10 mM glucose + 10 μM forskolin + 10 μM IBMX (F/I/G), 20 mM Gly-Leu (G-L) or 1 μM phorbol meryistate acetate (PMA) (B). GLP-1 concentrations were measured at the end of the experiment in both the supernatant and cell lysate of each well. In (A), basal GLP-1 secretion is represented as the % of the total GLP-1 content released in 2 h under control conditions (*n* = 9 wells per column, comprising 3 wells each from 3 independent mice). In (B), secretion has been normalized to the basal secretory rate measured in control wells from the same culture (*n* = 9, as in (A)). *p < 0.05, ***p < 0.001 by 2-way ANOVA with post hoc Bonferroni test, performed on log transformed data.

We next evaluated whether the function of L-cells was modified by high fat feeding. GLP-1 secretion was assessed in small intestinal cultures from mice fed for 2 weeks on either HFD or chow (Fig. 3). Cultures from chow-fed mice had a basal secretory rate of 2.8% per 2 h, which was stimulated 3.5-fold by 10 mM glucose, 7.9-fold by 0.5% peptone, 1.6-fold by 100 μM linoleic acid, 20-fold by glucose/fsk/IBMX, 3.2-fold by 20 mM Gly-Leu, and 5.6-fold by 1 μM PMA. Cultures from HFD-fed mice exhibited an increased rate of basal GLP-1 release (5.0% per 2 h, cf. 2.8% in chow-fed animals, *p* < 0.05). The amplification of GLP-1 secretion above the basal rate by glucose, peptone, forskolin/IBMX and Gly-Leu was reduced in the HFD-fed group (Fig. 3) although absolute % secretory rates in the presence of different stimuli were similar in chow-fed and HFD-fed mice.

Basal and stimulated GLP-1 release from colonic cultures were not altered by placing mice on HFD for 2 weeks (data not shown). In mice fed for 16 weeks on HFD, however, basal GLP-1 secretion from colonic cultures was elevated ∼2-fold, from 2.4 ± 0.2% per 2 h in chow fed mice (*n* = 12 wells from *n* = 4 mice) to 5.6 ± 0.9% in HFD-fed mice (*n* = 9 wells from *n* = 3 mice, *p* = 0.0005).

### 3.4 Effect of HFD on L-cell gene expression

**Fig. 4:**
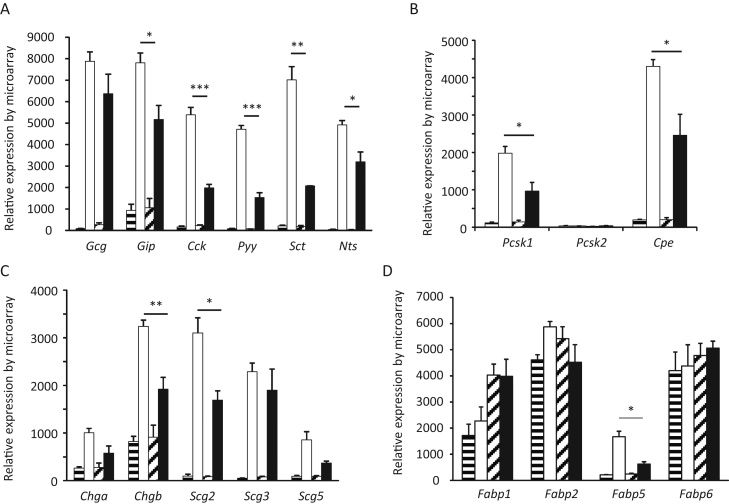
Effect of HFD on L-cell gene expression. Small intestinal L-cells and control non-L-cells were purified by FACS from mice fed for 16 weeks on chow or HFD, and analysed using Affymetrix ST1.0 microarrays. Data are represented as the RMA intensity. Non-L-cells are represented by striped bars (chow, horizontal, *n* = 3; HFD, diagonal, *n* = 4), and L-cells by filled bars (chow, white, *n* = 3; HFD, black, *n* = 3). Expression of gut hormones (A), prohormone processing enzymes (B), granins (C) and fatty acid binding proteins (D) are illustrated. Data are depicted as the mean and 1 SEM. Expression in L-cells from chow and HFD mice was compared by Student's *t*-test: *p < 0.05, **p < 0.01, ***p < 0.001.

The effect of HFD on gene expression in L-cells was evaluated using GLU-Venus mice that were fed on a HFD or chow diet for 16 weeks. HFD-feeding in this group resulted in a significant increase in body weight as well as fat-pad weight. Small intestinal L-cells and non-L-cells from 3 chow-fed and 4 HFD-fed mice were separated by FACS-sorting and subjected to mRNA microarray analysis. Intensity values calculated by RMA analysis indicate the relative expression of individual genes in L-cells and non-L-cells, and were compared in both chow and HFD-fed cohorts (Fig. 4).

*Gcg* expression was not different between L-cells from HFD and chow-fed mice. This was expected, as L-cells were collected based on their fluorescence of Venus driven by the *gcg* promoter. Expression of mRNAs for the gut hormones *Gip*, *Cck*, *Pyy*, secretin (*Sct*) and neurotensin (*Nts*) were significantly reduced in small intestinal L-cells from HFD-fed mice (Fig. 4A). Corresponding with these reduced mRNA levels for gut hormones, L-cells from HFD mice also exhibited lower expression of the prohormone processing enzymes *Pcsk1* (prohormone convertase 1/3, *p* = 0.026) and *Cpe* (carboxypeptidase E, *p* = 0.034) (Fig. 4B), as well as members of the granin group, particularly chromogranin B (*Chgb*, *p* = 0.009) and secretogranin 2 (*Scg2*, *p* = 0.019) (Fig. 4C).

**Fig. 5:**
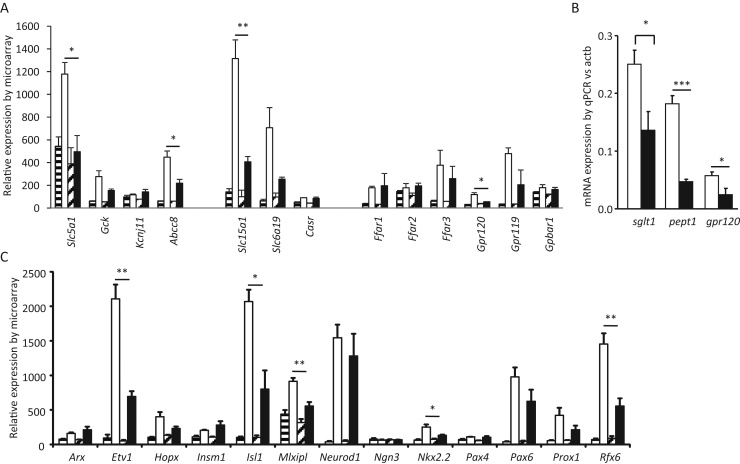
Effect of HFD on L-cell expression of nutrient sensing genes and transcription factors. Small intestinal L-cells and control non-L-cells were purified by FACS from mice fed for 16 weeks on chow or HFD, and analysed using Affymetrix ST1.0 microarrays. Data in (A) and (C) are represented as the RMA intensity. Non-L-cells are represented by striped bars (chow, horizontal, *n* = 3; HFD, diagonal, *n* = 4), and L-cells by filled bars (chow, white, *n* = 3; HFD, black, *n* = 3). (B) Gene expression in L-cells analysed by qRT-PCR, analysed as ΔCT data (actb-gene of interest) and transformed by 2^ΔCT^ for illustration: chow, white, *n* = 4; HFD, black, *n* = 4. In all panels, expression in L-cells from chow and HFD mice was compared by Student's *t*-test: *p < 0.05, **p < 0.01, ***p < 0.001.

To investigate whether the reduced amplification of GLP-1 secretion by nutrients might correlate with lower expression of nutrient-sensing machinery, we examined expression in L-cells of candidate sensory machinery for glucose (*Slc5a1*, *Kcnj11*, *Abcc8* and *Gck*), fatty acids (*Gpr120*, *Ffar1*), and oligopeptides (*Slc15a1*, *Casr*). Expression of *Slc5a1* (*sglt1*, *p* = 0.017), *Abcc8* (*sur1*, *p* = 0.024), *Slc15a1* (*pept1*, *p* = 0.006) and *Gpr120* (*p* = 0.017), were significantly lower in L-cells from mice on HFD than chow (Fig. 5A). The lower expression of *Slc5a1*, *Slc15a1* and *Gpr120* in HFD L-cells was also confirmed by qRT-PCR (Fig. 5B).

To confirm that the data do not represent a global reduction of gene expression in L-cells from HFD-fed mice, we examined expression of members of the fatty acid binding protein family. *Fabp1*, *2* and *6* were found to be ubiquitously expressed in the different gut cell populations, whereas *fabp5* expression was highly enriched in L-cells (Fig. 4D). Expression of the non-L-cell enriched FABPs (1, 2 and 6) was high in both L-cells and non-L-cells and was unaffected by HFD. Expression of *Fabp5*, by contrast, was enriched in L-cells compared with control cells, and was significantly lower in L-cells from mice on HFD (*p* = 0.01). Our data therefore suggest that feeding mice with HFD results in reduced expression in L-cells of many genes that determine the characteristics and function of an L-cell.

We further examined whether the down-regulation of L-cell genes is accompanied by changes in expression of transcription factors known to be involved in determination of the enteroendocrine-cell lineage. As shown in Fig. 5C, L-cells from mice on HFD exhibited a significantly reduced expression of *Etv1* (*p* = 0.003), *Isl1* (*p* = 0.017), *Mlxipl* (*p* = 0.009), *Nkx2.2* (*p* = 0.045) and *Rfx6* (*p* = 0.009).

## 4 Discussion

Our data show that mice fed with a HFD exhibited changes in their gut endocrine system compared with mice on chow diet. There were small changes in enteroendocrine cell number, altered hormone secretion, and reduced expression in L-cells of a number of genes that determine the properties and function of an L-cell.

Examination of global gene expression in tissue homogenates from different regions of the guts of mice fed for only 2 weeks on HFD revealed changes in mRNAs for gut hormones, particularly in the colon. The numbers of mice used for these experiments were, however, small and the data do not indicate whether the enteroendocrine cell number has changed, or whether the changes represent alterations in hormonal expression within EECs. FACS analysis with immuno-staining was therefore performed to quantify EECs in different regions of the gut and to confirm the production of gut hormones at the peptide level. This revealed that the number of L-cells was lower in the colons of HFD-fed mice, corresponding with the reductions in gene expression observed in the whole tissue homogenates. By FACS analysis it also appeared that the hormonal signature of colonic L-cells was shifted, with L-cells from the fat-fed mice exhibiting reduced CCK production and a tendency for increased PYY production. In the small intestine, by contrast, L-cell numbers did not appear to be affected by high fat feeding, but we observed a reduced number of GIP-stained cells.

As it has been reported that there are sufficient L-cells in the proximal small intestine to account for post-prandial hormone release [12], we examined whether L-cell transcriptomics and hormone release were altered in small intestinal L-cells. A large number of transcriptomic changes were observed in L-cells from mice fed on HFD, with a significant down regulation of expression of mRNAs encoding L-cell specific transcription factors, enteroendocrine hormones, prohormone processing enzymes, granins and nutrient sensing machinery. The data do not appear to represent either a global reduction in gene expression in L-cells, nor contamination of the FACS-purified L-cell pool by non-L-cells. We therefore conclude that the L-cells in mice fed HFD had impaired production of many genes required for the normal function of an enteroendocrine cell. This coincided with reduced expression of a number of EEC transcription factors. *Isl1*, *Nkx2.2* and *Rfx6* are transcription factors well known to play a role in the EEC lineage [13], [14], [15]. As *Rfx6* has been reported previously to promote *Gip* expression [15], the reduction in *Rfx6* might account for our observation of reduced *Gip* mRNA in L-cells. *Etv1* and *Mlxipl* (encoding CHREBP) were identified in our previous analysis of transcription factors enriched in L-cells [16]. The reduced expression in HFD L-cells of *Slc5a1*, *Abcc8*, *Slc15a1* and *Gpr120* would suggest that L-cells from mice on HFD might exhibit reduced nutrient responsiveness [17], [18], [19]. Indeed the changes in gene expression corresponded with an apparent reduced responsiveness of GLP-1 release to L-cell secretagogues in primary small intestinal cultures. Basal GLP-1 release, by contrast was elevated both in small intestinal cultures of mice fed with HFD for 2 weeks, and in colonic cultures of mice fed on HFD for 4 months. It is unclear why the basal rate of GLP-1 release was increased in the HFD-fed models, but we have observed a similar finding in ob/ob mice fed on chow (AMH, FG, FR unpublished observations). Whilst we cannot exclude the possibility that the L-cells from HFD and ob/ob mice are more fragile, resulting in the lysis of a few cells during the incubation period that would mimic a high basal secretory rate, we believe it is more likely that an imbalance of second messenger signaling pathways underlies this increased rate of basal secretion in the absence of added nutritional stimuli.

Our data showed that substantial changes in the properties of L-cells arose when mice were fed on a HFD. Compared with chow, the HFD we used contained a higher percentage of fat (60%) and free sugars, and no fiber. We cannot therefore be certain which of these dietary macronutrient changes was responsible for the alterations in L-cell gene expression. Diets rich in fat and sugar and low in fiber are, however, commonly consumed by humans in the western world. Our data would suggest that such diets may have adverse effects on our gut hormones. Whether humans who eat a diet rich in fat and sugar for prolonged periods exhibit increased basal GLP-1 release and lowered responsiveness to food ingestion will be interesting to examine in the future. A reduced post-prandial elevation of GLP-1 would, however, tend to lower the body's ability to signal satiety, and could result in a vicious cycle of over-eating. Resetting gut hormone responsiveness by a period on a healthy diet might, therefore be a strategy to increase post-prandial satiety and tackle obesity.
